# Face identity coding in the deep neural network and primate brain

**DOI:** 10.1038/s42003-022-03557-9

**Published:** 2022-06-20

**Authors:** Jinge Wang, Runnan Cao, Nicholas J. Brandmeir, Xin Li, Shuo Wang

**Affiliations:** 1 Lane Department of Computer Science and Electrical Engineering, West Virginia University, Morgantown, WV 26506 USA; 2 Department of Neurosurgery, West Virginia University, Morgantown, WV 26506 USA; 3 Department of Radiology, Washington University in St. Louis, St. Louis, MO 63110 USA

**Keywords:** Perception, Neural encoding

## Abstract

A central challenge in face perception research is to understand how neurons encode face identities. This challenge has not been met largely due to the lack of simultaneous access to the entire face processing neural network and the lack of a comprehensive multifaceted model capable of characterizing a large number of facial features. Here, we addressed this challenge by conducting in silico experiments using a pre-trained face recognition deep neural network (DNN) with a diverse array of stimuli. We identified a subset of DNN units selective to face identities, and these identity-selective units demonstrated generalized discriminability to novel faces. Visualization and manipulation of the network revealed the importance of identity-selective units in face recognition. Importantly, using our monkey and human single-neuron recordings, we directly compared the response of artificial units with real primate neurons to the same stimuli and found that artificial units shared a similar representation of facial features as primate neurons. We also observed a region-based feature coding mechanism in DNN units as in human neurons. Together, by directly linking between artificial and primate neural systems, our results shed light on how the primate brain performs face recognition tasks.

## Abstract (web summary)

A deep neural network that was trained to identify celebrity faces can be used to model primate face recognition, and reveals the importance of identity-selective neural units in face recognition.

## Introduction

The ability to identify and recognize faces is one of the most important cognitive functions in social communications. Primates have a dedicated neural system to process faces. Neurons that are selectively responsive to faces (i.e., face-selective neurons) have been observed in a distributed network of brain areas, notably including the face patches in the temporal cortex^1^ (shown in monkeys) as well as the amygdala and hippocampus^2,3^ (shown in both monkeys and humans). In particular, there are two extreme hypotheses about how neurons encode and represent faces. The exemplar-based model posits that faces are represented by highly selective, sparse, but visually invariant neurons^4–7^. This model has been supposed by single-neuron recordings in the human amygdala, hippocampus, and other parts of the medial temporal lobe (MTL). The feature-based model posits that faces are represented by simultaneous activation of a broad and distributed population of neurons and each neuron responds to many pictures with similar basic features^8,9^. This model has been supported by single-neuron recordings in the monkey inferotemporal (IT) cortex^10–13^. Although exemplar-based and feature-based models are not mutually exclusive because both types of neurons have been observed in different brain regions, it remains unclear how to bridge these prior findings and how the brain transitions from one model to the other, which is largely due to the lack of simultaneous access to the activity of the entire face processing neural network (i.e., the whole population of neurons from all brain regions involved in face processing)^14^.

To address this limitation, in this study, we exploit the opportunity to analyze the activity of all units from an artificial neural network (ANN) dedicated to face recognition. Importantly, we were able to compare the results directly with recordings from the primate neurons, which will shed light on how the primate brain encode face identities. Rapid advances in computer vision and the development of deep neural networks (DNNs) have provided an unprecedented opportunity to study face recognition and representation^15^. DNNs can help researchers to understand the functional architecture of the primate brain and test the computational benefits of fundamental organizational features for the visual system^16^. For example, within a class of biologically plausible hierarchical neural network models, there is a strong correlation between a model’s categorization performance and its ability to predict single-neuron responses from the IT cortex^17^. In addition, both human neuroimaging studies^18^ and intracranial electroencephalogram (EEG) studies^19^ have shown that features in DNNs can be represented in the human brain, which in turn can explain our ability to recognize individual faces. Recent studies in monkeys have shown that images synthesized by DNNs can control neural population activity^11,12^. Using natural face stimuli and face features extracted from DNNs, our recent work has shown that neurons in the human MTL encode visually similar identities^20^.

This study continues from our recent work showing feature-based encoding of face identities in the human MTL using DNN-extracted visual features^20^. The motivation for the present study is largely two-fold. First, the sparse coding hypothesis for face recognition has been inspected recently under the framework of DNN. It has been shown that identity, gender, and viewpoint information all contributes to individual unit responses^21,22^, similar to the neuronal coding of facial attributes in the primate brain^9,20,23^. Experimentally, it is difficult to comprehensively characterize the tuning properties of neurons from the human brain to a large number of facial attributes; therefore, in this study, we will conduct in silico experiments to probe the tuning properties of DNN units using a diverse array of stimuli, which will in turn shed light on the tuning properties of human neurons. Second, several lines of research in DNN (e.g., dropout^24^, neural architecture search^25^) have shown supporting evidence about the so-called lottery ticket hypothesis^26^ (i.e., among all possible fully connected and feedforward architectures, the winning ticket is a sparse subnetwork). This hypothesis has important biological implications into both energy efficiency and generalization properties, but there is still a missing parallel and convergent connection between the sparse coding hypothesis in neuroscience^27^ and the lottery ticket hypothesis in deep learning^26^. To fill in this gap, in this study, we used a DNN as a proxy model for studying the tuning properties of neurons that encode face identities. Our present study will directly compare and link the sparse coding of face identities between human neurons and DNN units.

## Results

### Identity-selective DNN units

**Fig. 1:**
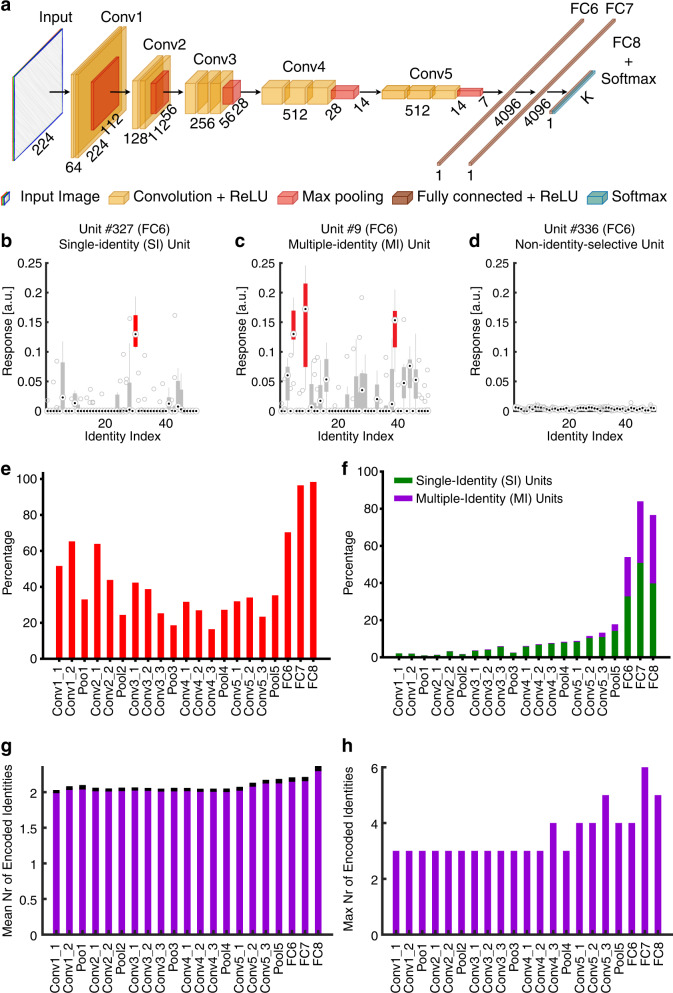
Identity-selective units in a pre-trained VGG-16 deep neural network (DNN). **a** Structure of the VGG-16 DNN. The convolutional neural network (CNN) consisted of a feature extraction section (13 convolutional layers) and a classification section (three fully connected (FC) layers). The feature extraction section was consistent with the typical architecture of a CNN. A 3 × 3 filter with 1-pixel padding and 1-pixel stride was applied to each convolutional layer, which followed by rectified linear unit (ReLU) operation. Every convolutional block was followed by a max-pooling operation with a stride of two pixels. There were three FC layers in each classification section: the first two had 4096 channels each, and the third performed a *K*-way classification. Each FC layer was followed by a ReLU and 50% dropout to avoid overfitting. A nonlinear Softmax operation was applied to the final output of VGG-16 network to make the classification prediction of 50 identities. **b** An example of a single-identity (SI) unit. **c** An example of a multiple-identity (MI) unit. **d** An example of a non-identity-selective unit (i.e., the unit did not encode any particular identities). Shown are responses of DNN units to 50 identities (500 faces in total; 10 faces per identity) in arbitrary units (a.u.). On each box, the central mark is the median, the edges of the box are the 25th and 75th percentiles, and the whiskers extend to the most extreme data points the algorithm considers to be not outliers. Encoded identities are shown in red. **e** Percentage of identity-selective units for each DNN layer. **f** Percentage of single-identity (SI; green) and multiple-identity (MI; purple) units for each DNN layer. **g** The average number of identities encoded by MI units. **h** The maximum number of identities encoded by MI units.

We used 500 natural face images of 50 celebrities (10 faces per identity) to elicit response from a pre-trained deep neural network (DNN) VGG-16 trained to recognize faces (see Fig. 1a for DNN architecture and Supplementary Fig. 1 for stimuli). We performed a fine-tuning on the top/output layer FC8 to ensure that the network was able to discriminate the identities used in the present study (see “Methods” for details). The pre-trained DNN had an accuracy of 94.2 ± 2.3% (mean ± SD across identities) in identity recognition after fine-tuning. Note that to make our results more comparable to the literature, we used the original VGG network without any fine-tuning for further analyses.

We identified a subset of DNN units that showed a significantly unequal response to different identities (one-way ANOVA of activation for each DNN unit: *P* < 0.01; Supplementary Fig. 2a), and we refer to this population of units as identity-selective units (see Fig. 1b–d for examples and Fig. 1e–g for the summary). There were identity-selective units in every layer (Fig. 1e). On the one hand, the high proportion of identity-selective units in earlier DNN layers (Fig. 1e) suggested that simple facial features (e.g., higher/lower contrast, more curves, more wrinkles, or more colorful makeup) could discriminate face identities. On the other hand, the higher percentage of identity-selective units in the later DNN layers (Fig. 1e) was likely because the later DNN layers were closer to the output (note that the output was the identity of the input face). Interestingly, we observed that a substantial amount of units were selective to multiple identities (referred to here as multiple-identity [MI] units; Fig. 1c, f, g, h; see “Methods” and Supplementary Fig. 2a for selection procedure), consistent with prior studies with direct recordings from single neurons in the human brain^20,28^. Compared to units that were selective to a single identity (referred to here as single-identity [SI] units; Fig. 1b, f), the percentage of MI units increased in the later DNN layers (Fig. 1f). Furthermore, the average number (Fig. 1g) and maximum number (Fig. 1h) of identities encoded by MI units showed that the receptive fields of the MI units increased from earlier layers to later layers. Lastly, some identities were encoded by more SI and MI units than the other identities (Supplementary Fig. 2b).

### Identity-selective DNN units demonstrated generalized selectivity to face identities

**Fig. 2:**
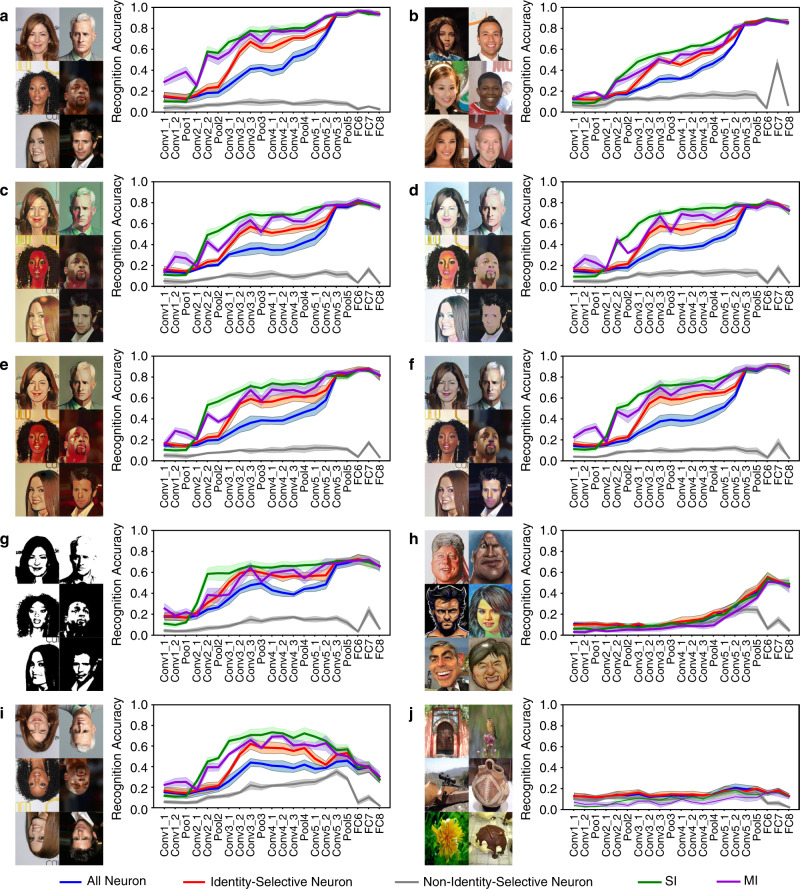
Selectivity properties of identity-selective units. **a** Original faces used to identify identity-selective units. **b** Faces from a different set of 50 identities randomly selected from the CelebA database. **c** Original faces in the cartoon style Hayao. **d** Original faces in the cartoon style Hosoda. **e** Original faces in the cartoon style Paprika. **f** Original faces in the cartoon style Shinkai. **g** Original faces in the Mooney style. **h** A different set of celebrity caricature faces. **i** Original faces in inversion. **j** A set of non-face objects selected from the ImageNet stimuli. Identity recognition accuracy is shown for each deep neural network (DNN) layer. Error shade denotes one standard deviation across five-fold cross-validation. Blue: all units from each DNN layer. Red: identity-selective units. Gray: non-identity-selective units. Green: single-identity (SI) unit. Purple: multiple-identity (MI) units.

We first analyzed the selectivity properties of identity-selective units. We used a support vector machine (SVM) to assess to what extent units from a specific DNN layer could distinguish the input stimuli. First, with the original stimuli used to select identity-selective units, we found that the discriminability of all DNN units for face identities increased in the later DNN layers (Fig. 2a). This was expected because the later DNN layers were closer to the output and contained more information about face identities. Notably, such discriminability was primarily driven by identity-selective units; and non-identity-selective units alone could not discriminate against different identities (Fig. 2a). Interestingly, identity-selective units had even better discriminability than all units in the earlier DNN layers (Fig. 2a); and SI and MI units had even better discriminability than all identity-selective units (Fig. 2a; also note that SI units had slightly better discriminability than MI units). It is worth noting that although the last five layers (Conv5_3, Pool5, FC6, FC7, and FC8) had a very different percentage of identity-selective units (Fig. 1e), the identity discrimination performance was similar across these layers (Fig. 2a; similarly for other stimuli as shown in Fig. 2), indicating that the layer Conv5_3 might have already contained sufficient information for identity discrimination. The similar accuracy across all last five layers and unit types (all vs. identity-selective vs. SI vs. MI) indicated that the information for identity discrimination was already saturated.

Second, we found that the DNN could discriminate identities in a different set of celebrity faces (Fig. 2b), as well as the original celebrity, faces transformed to various cartoon styles (Fig. 2c–f), and the response profile was similar to that with the original stimuli (although cartoon faces had an overall reduced discrimination accuracy): identity-selective units primarily drove the discrimination and had even better discriminability than all units in the earlier DNN layers whereas non-identity-selective units could not discriminate identities in all these tests. Within identity-selective units, SI and MI units primarily drove the discrimination.

Third, we found that identity-selective units could still discriminate face identities even with very limited information in faces (Fig. 2g). Although the accuracy was reduced to discriminate these low-information two-tone Mooney faces (generated from the original celebrity faces), a similar pattern of response was observed with identity-selective units (especially SI and MI units) primarily driving the discrimination.

Fourth, we found that identity-selective units could discriminate a different set of celebrity caricature faces in an exaggerated cartoon style (Fig. 2h). Although the overall accuracy was reduced, identity-selective units still played the dominant role in discriminating the identities and non-identity-selective units did not contribute to the discrimination. It is also worth noting that identity-selective units (including SI and MI units) as well as all units could not discriminate face identities any more in earlier layers (Fig. 2h).

Fifth, we found that the DNN could still discriminate inverted faces (Fig. 2i), although the accuracy was reduced, consistent with impaired discrimination of inverted face in humans^29^. Such discrimination was again driven by identity-selective units (especially SI and MI units). However, we found that the DNN (all units, identity-selective units, SI units, MI units, and non-identity-selective units) could barely discriminate non-face object categories (Fig. 2j). Therefore, the response could only be generalized within faces.

Lastly, we conducted the following control analyses. (1) We derived similar results when we equated the number of units per layer when comparing identity-selective and non-identity-selective units (Supplementary Fig. 3), since classification performance could depend on the number of units (features). (2) We found that DNN units selective to identities from one race (e.g., Caucasian) could also discriminate face identities from other races, suggesting that the DNN and identity-selective units had cross-race generalizability. (3) We found that DNN units selective to identities from one gender (e.g., male) could also discriminate face identities from the other gender, suggesting that the DNN and identity-selective units had cross-gender generalizability. (4) We found that combined SI and MI units (i.e., a subset of identity-selective units selected by an additional criterion that the response for certain identities stood out from the global mean response; see “Methods” and Supplementary Fig. 2a) demonstrated even stronger discriminability of face identities (Supplementary Fig. 2c). (5) Although most of our stimuli were frontal faces (Supplementary Fig. 1), we confirmed that the DNN could well discriminate profile faces as well (Supplementary Fig. 4a, b). (6) Using a VGG network pre-trained for ImageNet object stimuli^30,31^, we confirmed that the VGG network could discriminate the non-face object categories (Supplementary Fig. 4c; see Fig. 2j for a comparison). (7) We employed different DNNs and found that our findings could generalize to other DNNs (Supplementary Fig. 5; note that as expected face-recognition performance was reduced in some DNNs that were not trained for face recognition).

Together, our results showed that identity-selective units played a general and critical role in discriminating face identities under various circumstances, whereas non-identity-selective units could discriminate face identities in none of the circumstances. Therefore, our results suggested that although the discriminability varied as a function of the level of information contained in the stimuli, a subset of DNN units were consistently involved in the face identity discrimination and these units demonstrated generalized selectivity to face identities.

### DNN visualization explained the role of identity-selective units in face recognition

**Fig. 3:**
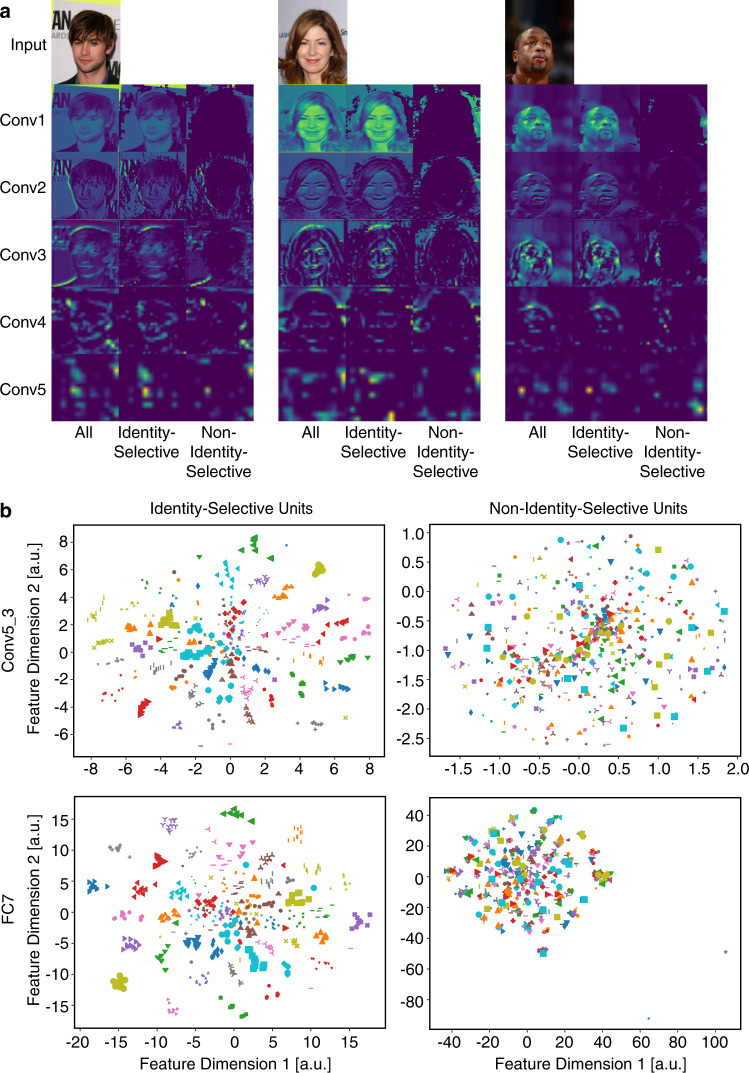
Visualization of the deep neural network (DNN). **a** Example activation maps for a subset of DNN layers. Left: all units. Middle: identity-selective units. Right: non-identity-selective units. **b** Visualization in the t-SNE space for DNN layers Conv5_3 (upper row) and FC7 (lower row). Each color-shape combination represents a face identity (ten faces per identity). The feature dimensions are in arbitrary units (a.u.). Left column: identity-selective units. Right column: non-identity-selective units.

We next visualized the response of identity-selective vs. non-identity-selective units in order to understand why identity-selective units but not non-identity-selective units played a general and critical role in face recognition. First, we found that identity-selective units indeed corresponded to the critical visual features of the stimuli such as the eyes, nose, and mouth (Fig. 3a). Second, when we constructed a two-dimensional stimulus feature space using t-distributed stochastic neighbor embedding (t-SNE) feature reduction for each DNN layer, we found that face identities clustered in the feature space constructed by identity-selective units but not non-identity-selective units (Fig. 3b), confirming that identity-selective units could discriminate face identities. Similar results were derived if we constructed a three-dimensional feature space or used different perplexity parameters for t-SNE (balance between local and global aspects of the data). We could also replicate our results in the full-dimensional space of the DNN. Together, DNN visualization revealed that identity-selective units encoded critical stimulus features so that they embodied a general discriminability of face identities.

### Lesion and perturbation of the network

We next investigated how critical the identity-selective units as well as the trained network weight structure were to discriminate face identities by lesioning and perturbing the network.

**Fig. 4:**
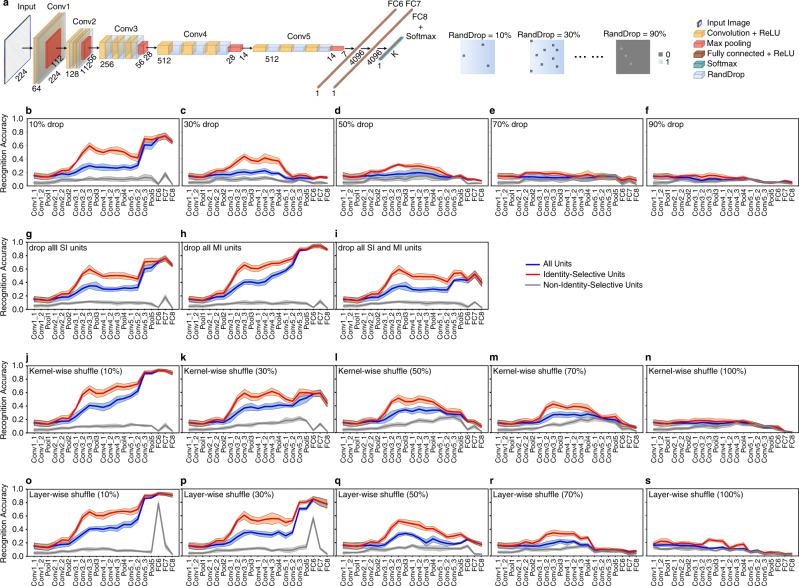
Manipulation of the deep neural network (DNN). **a** Illustration of how random dropout of DNN units was performed. We added a binary mask following every convolutional layer in the original DNN architecture to randomly deactivate a subset of DNN units. The percentage of dropped DNN units was controlled by the percentage of zeros in the binary mask and varied from 10 to 90%. **b**–**f** Recognition accuracy following a random dropout of DNN units. **g**–**i** Recognition accuracy following a complete dropout of (**g**) SI units, (**h**) MI units, and (**i**) combined SI and MI units. **j**–**n** Recognition accuracy following kernel-wise shuffle. **o**–**s** Recognition accuracy following layer-wise shuffle. Legend conventions as in Fig. 2.

First, following each convolutional layer, we added a “RandDrop” layer (i.e., a binary mask applied to the preceding layer; Fig. 4a) to randomly set a subset of DNN units to be 0, which partially lesioned the network. Indeed, we found that identity recognition accuracy decreased as a function of increasing lesion amount (Fig. 4b–f). With a small amount (10%) of information loss (Fig. 4b), the network could still well discriminate face identities and only had a small decrease in performance compared to the intact network (Fig. 2a; the decrease in performance was primarily in later layers). When 30–50% of DNN units were dropped (Fig. 4c, d), only earlier layers had a comparable performance as the intact network but later layers had a significant decrease of performance. When 70–90% of DNN units were dropped (Fig. 4e, f), the network could not perform identity discrimination any more. Notably, in all these cases, identity-selective units still played the dominant role in discriminating the identities and non-identity-selective units did not contribute to the discrimination. It is also worth noting that we dropped the same percentage of units for every layer, so information loss would accumulate in later layers. Interestingly, we found that dropping units from a single layer had only limited impact on recognition performance in subsequent layers (Supplementary Fig. 6; 30% of units were dropped; only dropping units from the very first layer Conv1_1 impacted performance in subsequent layers; no re-training was involved), suggesting that the network had great plasticity.

Furthermore, we investigated the impact of SI and MI units on identity discrimination by dropping these units (Fig. 4g–i). We found that when dropping SI units (Fig. 4g) and MI units (Fig. 4h) alone, the network could still well discriminate face identities, indicating that SI and MI units could well complement each other. The decrease in performance was primarily in the later layers (likely due to accumulation of information loss, as shown above), and the earlier layers had a comparable performance as the intact network (Fig. 2a). Dropping all SI units (Fig. 4g) had a similar impact as dropping 10% of all units (Fig. 4b), whereas dropping all MI units (Fig. 4h) had a less impact and led to a performance comparable to the intact network (Fig. 2a), indicating that MI units were less important compared to SI units in identity discrimination (see also Fig. 2 where SI units had a slightly better identity discriminability compared to MI units). As expected, when we dropped both SI and MI units (Fig. 4i), the performance further decreased, leading to a performance comparable to dropping 30% of all units (Fig. 4c).

Second, we perturbed the network by breaking the optimal weight structure (i.e., connection between DNN units) derived from training and generated a random permutation of weights (i.e., connections between DNN units) to evaluate the impact of the model training on identity recognition. We employed two approaches. Kernel-wise shuffle randomly permuted the weights in a single kernel. Similar to the above dropout results, the DNN gradually lost the ability to discriminate face identities with increasing levels of kernel-wise shuffle (Fig. 4j–n). Unlike kernel-wise shuffle that only rearranged the weights within one kernel, layer-wise shuffle pooled the weights of all kernels from a layer and reorganized the weights to form new kernels. Again, we found that with increasing levels of layer-wise shuffle, the DNN’s ability to discriminate face identities decreased and was eventually abolished (Fig. 4o–s).

Together, our model manipulation suggested that identity-selective units, as well as the optimal DNN unit connections derived from training, were critical to face identity discrimination.

### Establishing the relationship between artificial DNN units and real monkey neurons

**Fig. 5:**
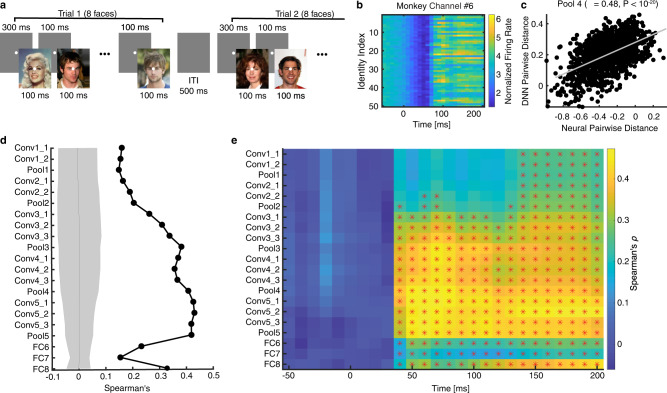
Comparison between the deep neural network (DNN) units and (real) monkey inferotemporal (IT) cortical neurons. **a** Task used to acquire neural responses from a monkey. In each trial, eight faces were presented for 100 ms each, followed by a fixed interstimulus interval (ISI) of 100 ms. There was a central fixation point of 300 ms at the beginning of each trial and there was an intertrial interval (ITI) of at least 500 ms following each trial. The central fixation point persisted through the trial. **b**, **c** An example multiunit-activity (MUA) channel showing a significant correspondence with the DNN feature space. **b** MUA to 50 identities, shown in 10 ms time bins. Time 0 denotes the stimulus onset. Firing rate was normalized to the average of the gray images (i.e., control stimuli). **c** Correlation between MUA pairwise distance and DNN layer Pool4 feature pairwise distance (see “Methods“). Each dot represents a face pair, and the gray line denotes the linear fit. **d** Correlation between pairwise distance in the monkey inferotemporal (IT) neuronal face space and pairwise distance in the DNN face space. Here, we used the mean firing rate in a time window 70 ms to 180 ms after stimulus onset as the response to each face, and we averaged the responses to ten faces for each face identity. We calculated the correlation using all identities. Solid circles represent a significant correlation (permutation test: *P* < 0.05, Bonferroni correction across layers). The shaded area denotes ±SD across permutation runs. **e** Temporal dynamics of correlation of pairwise distance between monkey neurons and DNN units (bin size = 40 ms, step size = 10 ms). Color coding indicates the magnitude of Spearman’s correlation. Asterisks (*) indicate a significant correlation in that bin (permutation test: *P* < 0.05, Bonferroni correction across time bins for each layer).

It has been suggested that DNNs share similarities with the primate visual cortex and can therefore help us better understand the sensory cortex^32^. Here, we explored the relationship between artificial DNN units and real primate neurons. We first analyzed whether the DNN had a similar encoding as monkey inferotemporal (IT) neurons. We used the same stimuli (500 natural face images of 50 celebrities) and recorded neuronal activity using two Utah arrays in the anterior and central IT cortex (see “Methods”) while the monkey performed a passive viewing task (Fig. 5a). We identified 53 multiunit activity (MUA) channels that showed sufficient internal consistency and we focused on these channels for further analysis.

We found that IT MUA not only showed face responsiveness (Fig. 5b) but also encoded the geometry of DNN layers (Fig. 5c). To formally quantify this result at the group level, we used DNN units to construct a face space and correlated that with the IT neuronal face space using a distance metric (see “Methods“). Using pairwise activation similarities^19^, we found that the pairwise distance from the intermediate to later DNN layers significantly correlated with the neuronal pairwise distance from the monkey IT cortex (Fig. 5d; nonparametric permutation test: *P* < 0.05, Bonferroni correction), suggesting that the population of DNN units encoded the geometry of the face space similarly as monkey IT neurons. We further investigated the temporal dynamics of the correspondence between face spaces and found a strong correlation starting from ~50 ms after stimulus onset (Fig. 5e; permutation test: *P* < 0.05, Bonferroni correction across time bins for each DNN layer; note that a moving time window was used), consistent with the response latency of monkey IT neurons^33,34^. Therefore, with our direct recordings from the monkey IT cortex using the same stimuli, we showed that the DNN shared a similar encoding of faces as the monkey IT cortex.

### Establishing the relationship between artificial DNN units and real human neurons

**Fig. 6:**
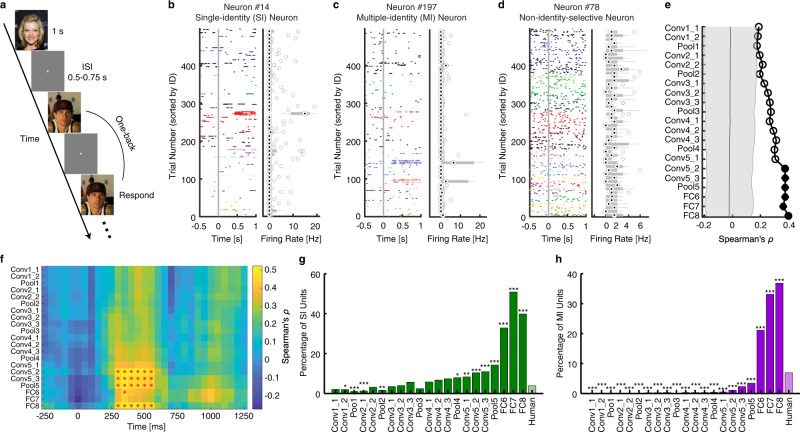
Comparison between the deep neural network (DNN) units and (real) human medial temporal lobe (MTL) neurons. **a** Task used to acquire single-neuron responses from humans. We employed a one-back task, in which patients responded whenever an identical famous face was repeated. Each face was presented for 1 s, followed by a jittered interstimulus interval (ISI) of 0.5–0.75 s. **b**–**d** Neuronal responses to 500 faces (50 identities) shown in raster plots. **b** An example of a single-identity (SI) neuron. **c** An example of a multiple-identity (MI) neuron. **d** An example of a non-identity-selective neuron (i.e., the neuron did not encode any particular identities). Trials are aligned to face stimulus onset (gray line) and are grouped by individual identity. On each box, the central mark is the median, the edges of the box are the 25th and 75th percentiles, and the whiskers extend to the most extreme data points the algorithm considers to be not outliers. **e** Correlation between pairwise distance in the human MTL neuronal face space and pairwise distance in the DNN face space. Here, we used the mean firing rate in a time window 250–1000 ms after stimulus onset as the response to each face, and we averaged the responses to 10 faces for each face identity. We calculated the correlation using the top ten identities that were most frequently encoded by MTL neurons. Solid circles represent a significant correlation (permutation test: *P* < 0.05, corrected by false discovery rate (FDR)^35^ for *Q* < 0.05) and open circles represent a non-significant correlation. The shaded area denotes ±SD across permutation runs. **f** Temporal dynamics of correlation of pairwise distance between human neurons and DNN units (bin size = 500 ms, step size = 50 ms). Color coding indicates Spearman’s correlation coefficient. Asterisks (*) indicate a significant correlation in that bin (permutation test: *P* < 0.05, FDR corrected across time bins for each layer). **g** Percentage of SI units in each DNN layer and comparison with SI neurons from the human MTL. **h** Percentage of MI units in each DNN layer and comparison with MI neurons from the human MTL. Asterisks indicate a significant difference in the percentage using *χ*^2^-test with Bonferroni correction for multiple comparisons. *P < 0.05, **P < 0.01, and ***P < 0.001.

The DNN performs the face-recognition task similarly as humans. Does the ensemble of DNN units share representational similarity with the ensemble of human neurons? In order to answer this question, we used the same stimuli (500 natural face images of 50 celebrities) and recorded from 667 neurons in the MTL (340 neurons from the amygdala, 222 neurons form the anterior hippocampus, and 105 neurons from the posterior hippocampus; firing rate >0.15 Hz) of 8 neurosurgical patients (23 sessions in total)^20^. Patients performed a one-back task (Fig. 6a; accuracy = 77.38 ± 4.94% [mean ± SD across sessions]) and they could well recognize the faces^20^. The responses of 76/667 neurons (11.39%) differed between different face identities in a window 250–1250 ms following stimulus onset and these neurons were the real human identity-selective neurons (see Fig. 6b–d, e.g., neurons). We grouped amygdala and hippocampal neurons as a single neuronal population (i.e., MTL neurons) for further analysis because they show very similar identity-selectivity responses^6,20^.

Similar to our analysis of IT MUA, we used DNN units to construct a face space and correlated that with the MTL neuronal face space using a distance metric (see “Methods”). We found that the pairwise distance from the later/top DNN layers significantly correlated with the neuronal pairwise distance from the human MTL (Fig. 6e; nonparametric permutation test: *P* < 0.05, corrected by false discovery rate (FDR)^35^ for *Q* < 0.05), suggesting that the population of DNN units encoded the geometry of the face space similarly as human MTL neurons. Notably, compared to IT neurons that had a drop in correspondence with DNN features from the later layers (Fig. 5d), MTL neurons had a smooth increase of correspondence and peaked at the top/output layer (Fig. 6e), consistent with the different processing stages along the ventral visual pathway. Here, we calculated the correlation between DNN units and human neurons using the ten identities that were most frequently encoded by MTL neurons, given the sparseness of MTL responses; but we derived a similar pattern of results using all 50 identities. We further investigated the temporal dynamics of the correspondence between face spaces and found a significant correlation in later DNN layers starting from 250 ms after stimulus onset (Fig. 6f; permutation test: *P* < 0.05, FDR corrected across time bins for each DNN layer), consistent with the response latency of human MTL neurons^36^.

In addition, we found that compared to the human MTL, the DNN had a significantly higher percentage of SI units in later layers (starting from the layer Pool4; Fig. 6g; *χ*^2^-test with Bonferroni correction for multiple comparisons). The DNN also had a significantly higher percentage of MI units in the layers FC6, FC7, and FC8 (Fig. 6h) but a significantly lower percentage of MI units in all other layers, where we did not expect to observe MI units because faces of the same identity were not yet clustered.

Together, using human single-neuron recordings we directly compared identity selectivity between artificial units and real human neurons. Our results have revealed a systematic correspondence between the two face-recognition systems.

### Region-based feature coding in DNN units and a mechanism underlying face recognition

How does the DNN transition from representing visual features (in earlier and intermediate layers) to representing identities (in later and output layers)? Inspired by the primate visual system, one possible mechanism is that earlier DNN layers encode the axes of a face space and provide information to later DNN layers, which encode a region in the high-level feature space and are selective to identities that fall in this region. This mechanism has been instantiated in the primate brain: IT neurons encode visual features and axes of the feature space^10–13,17^ (notably, the axes of our CelebA face space in the current study have been shown^20^), whereas MTL neurons are selective to specific face identities^6^. Our recent study has supported such transition by revealing a region-based feature coding by real human MTL neurons^20^, i.e., human MTL neurons encode a specific region in the feature space and are selective to identities that are clustered in this region. Here, we further compared between artificial and primate neural systems and explored whether DNN units also demonstrated region-based feature coding, which will provide critical insights into the computational mechanisms underlying face recognition.

**Fig. 7:**
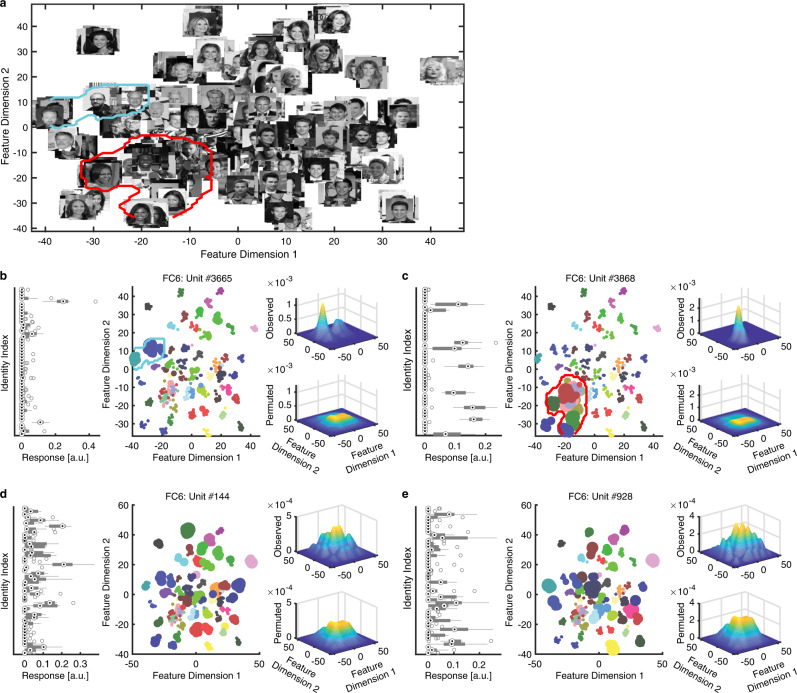
Region-based feature coding in DNN units. **a** The face feature space constructed by t-distributed stochastic neighbor embedding (t-SNE) for the DNN layer FC6. All stimuli are shown in this space in grayscale. **b**–**e** Example DNN units. **b**, **c** Two examples of MI units that demonstrated region-based feature coding (i.e., the units encoded a region in the feature space). **d** An example MI unit that did not demonstrate region-based feature coding (i.e., the encoded identities were not adjacent to each other in the feature space). **e** A non-identity-selective unit (i.e., the unit did not encode any particular identities). All examples were from the DNN layer FC6. Left: Response of DNN units to 50 identities (500 faces in total; ten faces per identity) in arbitrary units (a.u.). On each box, the central mark is the median, the edges of the box are the 25th and 75th percentiles, and the whiskers extend to the most extreme data points the algorithm considers to be not outliers. Middle: Projection of the DNN activation onto the feature space. Each color represents a different identity. The size of the dot indicates the level of activation. Right: Estimate of the spike density in the feature space. By comparing observed (upper) vs. permuted (lower) responses, we could identify a region where the observed response was significantly higher in the feature space. This region was defined as the tuning region of a unit (delineated by the red/cyan outlines).

We focused on the DNN layers FC6 and FC7, where faces of the same identity was clustered; and we primarily observed human MTL neurons demonstrating region-based feature coding in the face feature spaces from these layers^20^. Notably, the face feature spaces showed an organized structure: for example, Feature Dimension 2 represented a gender dichotomy, and darker skinned faces were clustered at the bottom left corner of the feature space (Fig. 7a). Indeed, we found that a large number of MI units in these layers (24.9% for FC6 and 36.5% for FC7) encoded identities that were adjacent in the feature space (see Fig. 7b, c), demonstrating region-based feature coding. We refer to this subpopulation of MI units as feature MI units. On the other hand, non-feature MI units did not have selective identities clustered in the feature space (Fig. 7d), and non-identity-selective units did not encode any particular identities (Fig. 7e).

**Fig. 8:**
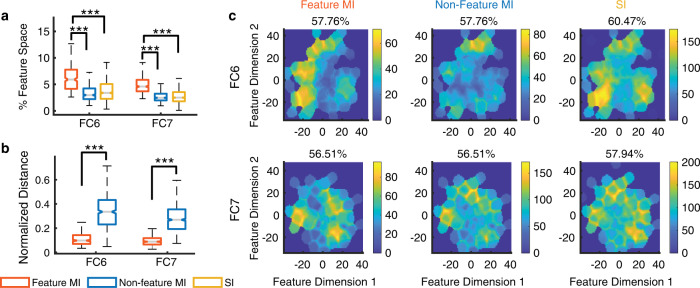
Summary of region-based feature coding for SI and MI DNN units. **a** Percentage of feature space covered by tuning regions of SI and MI units. Note that here we did not apply the threshold for minimal cluster size for SI and non-feature MI units in order to compare between different types of units. **b** Normalized distance between MI unit’s selective identities in the feature space. To be comparable for different layers, Euclidean distance was normalized by the maximum distance (i.e., diagonal line) of the feature space. On each box, the central mark is the median, the edges of the box are the 25th and 75th percentiles, and the whiskers extend to the most extreme data points the algorithm considers to be not outliers. Asterisks indicate a significant difference between feature MI units and non-feature MI units using two-tailed unpaired *t* test. ***P < 0.001. **c** The aggregated tuning regions of the population of units. Color bars show the counts of overlap between individual tuning regions. The numbers above the density map show the percentage of feature space covered by the tuning regions of the population of units.

At the population level, we found that the tuning region of an individual feature MI unit covered ~5–6% of the 2D feature space (Fig. 8a; note that when we calculated the tuning region, we adjusted the kernel size to be proportional to the feature dimensions such that the percentage of space coverage was not subject to the actual size of the feature space). In contrast, the response of an individual SI or non-feature MI unit covered a significantly smaller region in the feature space (Fig. 8a; two-tailed unpaired *t* test: *P* < 0.001 for all comparisons). As expected, the distance in the face space between encoded identities was smaller for feature MI units compared with non-feature MI units (Fig. 8b). As a whole, the entire population of DNN units covered ~55–60% of the feature space (Fig. 8c; some areas were encoded by multiple units), and the covered areas were similar for SI, feature MI, and non-feature MI units (Fig. 8c).

We conducted several control analyses to ensure that our findings were robust in regard to the construction of the feature space. (1) We derived similar results if we constructed a three-dimensional feature space, or used different perplexity parameters for t-SNE or kernel/cluster size parameters to detect a tuning region. (2) We derived similar results if we constructed the feature space using uniform manifold approximation and projection (UMAP) or principal component analysis (PCA). (3) We could replicate our findings using full DNN features, where the Euclidian distance between encoded identities was significantly smaller than that of non-encoded identities.

Together, we found that as neurons in the human MTL, DNN units also demonstrated region-based feature coding. Together with the default axis-based coding (i.e., DNN units encode a linear combination of features from a previous layer) and exemplar-based coding (i.e., DNN units output a face identity with visual invariance of the input images) in the DNN, our findings may provide an important mechanism that explains how the DNN transitions from representing visual features to representing identities and thus performs face-recognition tasks. Importantly, our results suggest that artificial and primate neural systems share similar computational mechanisms for face recognition.

## Discussion

In this study, we analyzed the response characteristics of a face-recognition DNN and found that identity-selective units in the DNN could generalize their discriminability to face identities shown in various styles as well as face identities that were not involved in the training. Visualization and manipulation of the DNN showed the importance of identity-selective units in face recognition. By establishing the coding similarity with real primate neurons, our study provided an important method to understand face coding in primates. Furthermore, by analyzing an artificial neural network dedicated to face recognition, we will be able to formulate hypotheses that can be validated in the primate brain.

In this study, we focused on analyzing the VGG-face model, which was pre-trained for face recognition and expected to contain identity-selective units. Using this functioning face-recognition model that is highly capable of processing face identity information enabled us to (1) test the generalizability of identity response to different categories of stimuli, (2) compare with primate visual systems, and (3) visualize and perturb the network to reveal the critical features for face identity discrimination. It is worth noting that here we also explored other DNN models (Supplementary Fig. 5) and found that our findings could generalize to other DNNs (although as expected face-recognition performance was reduced in some DNNs that were not trained for face recognition), consistent with a previous report surveying a large class of DNN models for face representation^37^. Interestingly, a recent study has even shown that face-selective units can emerge from an untrained DNN^38^. A future study will need to investigate whether identity-selective units can emerge from an untrained DNN.

We found that although cartoon faces had a decreased discriminability in general, they had a similar pattern of response as natural faces across DNN layers (Fig. 2a, c–f). Furthermore, we found that inverted faces elicited a similar response as upright faces in the early and middle DNN layers (Fig. 2a, i), suggesting that the DNN used similar information for both inverted and upright faces, a form of viewpoint invariance. However, in contrast to all upright faces that had increasing discriminability across layers, the discriminability decreased in later layers for inverted faces. Therefore, the impaired discriminability of inverted faces in humans may stem from neurons downstream in the visual processing stream^29^. Furthermore, consistent with our DNN lesion results (Supplementary Fig. 6), it has been shown that DNN units demonstrate distributed and sparse codes to represent different face attributes^22^. Lastly, our present results may depend on the hyperparameters used in the study (batch-norm vs. dropout, pooling, architecture, dataset size, etc.) and our results should be interpreted in the context of our DNN architecture and set of hyperparameters.

Although the SVM had substantially more features (i.e., DNN units used for classification) than observations (i.e., training faces), the high recognition accuracy of identity-selective units in testing suggested that our results could not be simply explained by overfitting. Furthermore, the low recognition accuracy of non-identity-selective units in testing provided specificity of our approach; and notably, later DNN layers had fewer units but a higher recognition accuracy. Interestingly, such overfitting also appears in the human brain as a large number of neurons are often simultaneously activated by a single percept; and the theory of backward feature correction can well explain such overfitting^39^. It is also worth noting that we derived similar results when we input the same number of identity-selective units and non-identity-selective units to the SVM (Supplementary Fig. 3), suggesting that the difference in encoded information between unit groups could not be attributed to the different number of input units.

We identified the DNN units critical for identity recognition, and the evolution of identity-selective units across DNN layers (Fig. 1e). The identity-selective units in the earlier layers primarily corresponded to image pixels containing information about faces, but with the increase of kernel size in later layers, identity-selective units encoded more holistic information about face identities. In particular, the fully connected layers utilized information from all units from the previous layers. Therefore, identity selectivity could not be solely attributed to the receptive field of the DNN units. It is worth noting that our present results were not about face selectivity (i.e., contrasting response between faces vs. objects) but identity selectivity (i.e., contrasting response between face identities, which does not require face selectivity^20^). However, we found that the response of face identity-selective units could well generalize within faces but barely generalize to non-face objects, consistent with a dedicated and specialized face perception system^1,38^. It is also worth noting that most monkey MUA channels showed strong face responsiveness (i.e., modulation by face onset; e.g., Fig. 5b), consistent with the previous studies^1^.

We observed both identity-selective units that were selective to a single identity (SI units) and identity-selective units that were selective to multiple identities (MI units), analogous to the SI neurons^6,20^ and MI neurons^20,28,40^ from the human brain. The SI units and MI units identified in the present study are also reminiscent of the concept cells of the exemplar-based model^7^. Concept cells primarily appear in the human medial temporal lobe, respond in a remarkably selective and abstract manner to particular persons or objects, may be crucial for memory formation^7^. Given that we here show computational similarities between the primate visual system and the DNN, future studies can benefit from the computational architecture of the DNNs and address two important questions: (1) how concept neurons arise computationally (i.e., the transition from representation of visual features to representation of concepts), and (2) how concept neurons create associations and transits between related concepts to form episodic memories.

We found that the DNN shared a similar coding with both monkey IT cortex and human MTL (see also ref. ^41^). Specifically, the intermediate to later DNN layers corresponded to the IT neuronal space whereas the later/top DNN layers corresponded to the MTL neuronal space, consistent with the ventral visual processing pathway in the primate brain^42,43^. In particular, in addition to the axis-based coding (i.e., DNN units encode a linear combination of features from a previous layer) and exemplar-based coding (i.e., DNN units output a face identity with visual invariance of the input images) in the DNN as observed in the IT cortex^10–13,17^ and MTL^6^, respectively, we confirmed the region-based coding in the DNN, which is an important mechanism that bridges the representation of visual features and the representation of identities. It is worthing that the correlation strength with DNN layers was different between the monkey (Fig. 5) and human (Fig. 6) visual systems, which was likely due to differences in recording method (Utah array vs. microwire), noise level in recordings, repetition (repeated vs. single) and duration (100 ms vs. 1 s) of stimulus presentation, recording location (cortical vs. subcortical), and spike sorting (multiunit vs. single-unit), so the correlation was not directly comparable between the IT cortex and the MTL. However, for each visual system, we found a significant correlation; and importantly, we found that the most strongly encoded DNN layer differed between brain areas in accordance with the ventral visual stream.

We found that identity-selective units had a general discriminability to face identities shown in various styles, consistent with feature-invariant coding of face identities by neurons in the human medial temporal lobe (MTL)^6,7^. Furthermore, identity selectivity could be generalized to face identities that were not involved in the training, similar to how memory is formed in the human brain^44^. Consistent with our prior findings that only a small proportion (~20%) of human neurons are involved in coding a certain task aspect, such as emotion content^45^, emotion subjective judgment^46^, attention^47,48^, task sequence^48^, visual selectivity^47^, eye movement^49^, social judgment^50^, as well as face identity^20^, in the present study we found a large population non-identity-selective DNN units that did not contribute to coding face identities. On the other hand, we quantitatively compared the proportion of SI and MI units/neurons between the DNN and human brain and found that the DNN in general had a higher proportion of identity-selective units than the human MTL (Fig. 6f, g). This was likely because the human MTL is involved in many aspects of cognitive functions whereas the DNN has only been optimized to recognize face identities. Notably, the distribution of identity-selective and non-identity-selective DNN units across layers may provide important insights into understanding the human visual processing stream, where our currently available technology does not allow simultaneous sampling of neurons along the entire visual processing stream.

Rapid advances in DNNs have offered new opportunities for studying face perception by providing computational proxy models. State-of-the-art DNNs such as the VGG-face^51^ and DeepFace^52^ have achieved excellent face-recognition performance and even outperformed humans. These DNNs are biologically inspired and therefore have the potential to successfully provide insight into the underlying mechanisms of brain functions, especially with respect to the perception and recognition of visual stimuli such as faces. Existing work at the intersection of DNNs and face perception can be broadly classified into two categories: face reconstruction (decoding models) and face recognition (encoding models). The former includes the reconstruction of faces from fMRI patterns^18,53^ (see refs. ^54,55^ for more general natural image reconstruction). The latter includes a flurry of literature on the convergent evolution of face spaces across DNN layers and human face-selective brain areas^19^, the neurally plausible efficient inverse graphics model for face processing^56^, and spontaneous generation of face recognition in untrained DNNs^38^. Our recent study showing feature-based encoding of face identities in the human MTL using DNN-extracted visual features^20^ also employed an encoding model.

DNNs have the following advantages to help us better understand visual processing in primates^32^. First, most previous studies of face space had to use computer-generated faces in order to parametrically vary the faces^10,57,58^ but DNNs are able to extract features from real human faces and subsequently manipulate these features to generate new unique faces while providing well-controlled stimuli to investigate differences in neural responses to feature changes. Second, DNNs have simultaneous access to the activity of the entire face processing neural network (i.e., the whole population of neurons from all brain regions involved in face processing)^14^, which is particularly useful to study visual processing pathways (e.g., how the brain transitions from one face coding model to another). Third, we are readily able to selectively manipulate DNN units to study a causal effect. These advantages have been embodied in our present study.

Our present study points to several future directions. First, most faces in the present study had a frontal view and were primarily emotionally neutral. A future study will further test faces from different angles (e.g., profile faces) and/or emotional faces. Second, an interesting future study will be to compare DNN lesion results with behavior of human brain lesion patients, who demonstrate impaired face perception^45^. The lesion approach can also help us better understand the functional segregation of face recognition^59^. Third, only one DNN architecture was examined in the present work; but more DNN architectures will need to be considered in order to further generalize our results. Lastly, region-based feature coding may also provide an account for object recognition (e.g., using the AlexNet) and visual selectivity (also observed in human MTL neurons): objects falling within the coding region of a neuron/unit may elicit an elevated response. Again, this mechanism applies to both artificial units and human neurons and needs to be tested in a future study.

A central challenge in cognitive neuroscience is to understand how the brain encodes faces. In particular, it remains largely unclear how visual experience, learning, and memory shape face perception and recognition. There are two competing hypotheses about the emergence of face-selective neurons. One hypothesis argues that face-selective neurons require visual experience to develop, and this hypothesis has been supported by fMRI studies in the monkey fusiform face areas^60^. The other hypothesis argues that face-selective neurons have an innate origin, and this hypothesis has been supported by studies from human infants and adults without visual experience of faces^61–64^. Along this line, future studies will be needed to explore if identity-selective units can spontaneously emerge from untrained neural network (but see also the lottery ticket hypothesis^26^) or neural networks trained with other image databases (e.g., ImageNet). Although the DNN used in the present study was a pre-trained artificial neural network, it demonstrated strong ability to generalize to new faces, comparable to the primate visual system. Importantly, in silico experiments allow us to test a large set of parameters and better control experimental conditions, which is often not feasible when directly working with human or animal subjects. Therefore, our present study not only highlights the importance of the direction of training and visual experience in shaping the neural response to face identities, but also provides a useful approach to test these hypotheses and reconcile previous findings.

## Methods

### Stimuli

We employed the following stimuli in this study (Fig. 2).

First, for the original stimuli, we used faces of celebrities from the CelebA dataset^65^, and we selected 50 identities with 10 images for each identity, totaling 500 face images. The identities were selected to include both genders and multiple races (see also Supplementary Fig. 1 and Fig. 7a).

Second, we selected another 500 faces from 50 different identities (10 images per identity) from the CelebA dataset as a testing set.

Third, we generated four versions of cartoon faces (Hayao, Hosoda, Paprika, Shinkai) of the original stimuli using CartoonGAN^66^.

Fourth, we generated Mooney faces by first transforming the original images into grayscale. We then filtered the images with a two-dimensional Gaussian smoothing kernel with standard deviation of 0.5. We lastly thresholded the images using a threshold determined for each individual face based on its luminance (threshold = mean luminance of the cropped image center − 0.03).

Fifth, we randomly selected 500 caricature faces of 50 identities (10 images per identity) from the IIIT-CFW dataset^67^.

Sixth, we randomly selected 500 non-face objects from 50 categories (10 objects per category) from the ImageNet database^68^.

### Deep neural network (DNN)

We used the well-known deep neural network (DNN) implementation based on the VGG-16 convolutional neural network (CNN) architecture^51^ (Fig. 1a; https://www.robots.ox.ac.uk/~vgg/software/vgg_face/). The inputs to the first convolutional layer were RGB images of fixed size (224 × 224 pixels). The images were passed through a stack of convolutional layers, where the filters were used with a very small receptive field (3 × 3 pixels, which was the smallest size to capture the notion of left/right, up/down, and center). The convolution stride was fixed to 1 pixel; and the spatial padding of the convolutional layer input was 1 pixel for 3 × 3 convolutional layers such that the spatial resolution was preserved after convolution. Spatial pooling was carried out by five max-pooling layers, which followed some of the convolutional layers. Max pooling was performed over a 2 × 2 pixel window, with a stride of two pixels. Three fully connected (FC) layers followed a stack of convolutional layers: the first two had 4096 channels each, the third performed 50-way classification and thus contained 50 channels (one for each identity). The final layer was the soft-max layer.

We fine-tuned the FC8 layer with the original CelebA stimuli to confirm that this pre-trained model was able to discriminate the identities and ensure that the pre-trained model was suitable as a feature extractor. Specifically, we modified the output layer to 50 units for our model. Two-thirds of the original CelebA stimuli were used as the training set and the remaining stimuli were used as the testing set. We used the Adam optimizer with an initial learning rate of 5 × 10^−4^ and we had 10 epochs in total. A learning rate scheduler was applied after each epoch with the gamma value set to 0.9 to facilitate the convergence of the loss function. To update the weights during fine-tuning, we computed the cross-entropy loss on random batches of four face images (scaled to 224 × 224 pixels) for backpropagation. We used five-fold cross-validation, which reached an accuracy of approximately 95%. Note that only the FC8 layer was fine-tuned and all the other layers were frozen. It is also worth noting that the VGG-16 was originally trained with 23 of the 50 identities involved in the present study (including Adam Levine, Bahar Soomekh, Betty White, Dana Delany, Dean Geyer, Eduardo Noriega, Hugh Jackman, Isla Fisher, John Slattery, Katherine Bailess, Kevin Hart, Logan Marshall-Green, Mario Lopez, Missi Pyle, Natalie Zea, Olivia Palermo, Rachel McAdams, Ron Perlman, Shawn Ashmore, Steven Soderbergh, Tim Gunn, Treat Williams, Zac Efron). However, we derived similar results when we excluded the identities involved in the original VGG-16 training (see also our generalization results with new identities in Fig. 2b).

To visualize the DNN response, we subsequently applied a t-distributed stochastic neighbor embedding (t-SNE) method to convert high-dimensional features into a two-dimensional feature space. t-SNE is a variation of stochastic neighbor embedding (SNE)^69^, a commonly used method for multiple class high-dimensional data visualization^70^. We applied t-SNE for each layer, with the cost function parameter (Prep) of t-SNE, representing the perplexity of the conditional probability distribution induced by a Gaussian kernel, set individually for each layer. We also used t-SNE to construct a face feature space so that we were able to investigate region-based feature coding for both DNN units and primate neurons^20^.

### Assessment of face-recognition accuracy

We used a support vector machine (SVM) to assess face-recognition accuracy for each group of DNN units from a specific DNN layer. We employed five-fold cross-validation: we randomly partitioned the stimuli into five equal portions, and in each run, four portions of the stimuli were used as the training dataset and the remaining portion of the stimuli was used as the test dataset. We used the python package Scikit-learn^71^ to build our radial basis function (RBF) kernel SVM classifier.

### DNN lesion and perturbation

By analogy with brain lesions, we designed a random-drop model to lesion the original VGG-16 network in order to understand how many DNN units were needed to discriminate face identities. We conducted two experiments. In the first experiment, following every convolutional layer, we included a binary mask for the preceding layer that randomly set a subset of DNN units to be 0. In the second experiment, we applied the binary mask to a specific layer only. Note that in both experiments, both identity-selective and non-identity-selective units were dropped, according to their proportions.

In addition, we perturbed the network by rearranging the weights in the model. We conducted two experiments. In the first experiment, kernel-wise shuffle, we randomly permuted the weights in a single kernel. Since the kernel size of the network was 3-by-3 for all layers, kernel-wise shuffle permuted the 9 weight values for each kernel. In the second experiment, layer-wise shuffle, we pooled the weights of all kernels from a layer and reorganized the weights to form new kernels. Note that in both experiments, both identity-selective and non-identity-selective units were shuffled.

### Neural recordings from a monkey

One male rhesus macaque (Macaca mulatta) was used in this study. All procedures conformed to local and U.S. National Institutes of Health guidelines, including the U.S. National Institutes of Health Guide for Care and Use of Laboratory Animals. All experiments were performed with the approval of the MIT Institutional Animal Care and Use Committee (IACUC).

The monkey passively viewed the original CelebA stimuli (Fig. 5a). In each trial, the monkey first viewed a white central fixation point (0.2 degrees of visual angle [DVA]) on a gray background for 300 ms to initiate a trial. Then, eight faces were presented for 100 ms each, each followed by a blank (gray) screen for an interstimulus-interval (ISI) of 100 ms. The central fixation point persisted through the trial, and fluid reward was given if the monkey successfully fixated through the entire trial. The intertrial-interval (ITI) of blank gray screen was at least 500 ms. We recorded 4155 trials in total, and we rejected 666 trials where the monkey broke the fixation (±2 DVA). For each round of presentation, we generated a random sequence for the 500 faces; and we used different sequences for different rounds of presentation. On average, each face was presented 55.7 ± 1.49 (mean ± SD) times. Note that we randomly inserted one gray image in each round of presentation as a control stimulus for baseline normalization.

The monkey was chronically implanted with two Utah arrays (Blackrock Microsystems) in the anterior and central inferotemporal (IT) cortex (see refs. ^33,34^ for details). Each array consisted of one 10-by-10 electrode grid with 96 active iridium oxide electrodes. Each electrode was 1.5 mm long with an inter-electrode distance of 400 μm. During each recording session, band-pass filtered (0.1 Hz to 7.5 kHz) neural activity was recorded continuously at a sampling rate of 20 kHz using Intan Recording Controller (Intan Technologies, LLC). We detected the multiunit spikes after the raw data were zero-phase band-pass filtered between 300 and 6000 Hz (Matlab ellip function, fourth-order with 0.1 decibel pass-band ripple and 40 dB stop-band attenuation), and we used multiunit activity (MUA) for analyses. A multiunit spike event was defined as the threshold crossing when voltage (falling edge) deviated by more than three times the standard deviation of the raw voltage values. We estimated internal consistency for each channel using a standardized image set that was run before the recording session on the same day and we accepted 53 MUA channels (from two arrays) that showed sufficient internal consistency (>0.6). Consistent with previous studies^33,34^, we used the mean firing rate in a time window 70 ms to 180 ms after stimulus onset as the response to each face. We averaged the response from repeated presentations for each face.

### Single-neuron recordings in human neurosurgical patients

To acquire the neuronal response from humans, we conducted single-neuron recordings from five neurosurgical patients (16 sessions in total). All participants provided written informed consent using procedures approved by the Institutional Review Board of West Virginia University (WVU). The detailed procedure has been described in our previous study^20^. Briefly, we employed a 1-back task for the original CelebA stimuli (Fig. 6a). In each trial, a single face was presented at the center of the screen for a fixed duration of 1 sec, with a uniformly jittered intertrial interval (ITI) of 0.5–0.75 s. Patients pressed a button if the present face image was identical to the immediately previous image. Each face was shown once unless repeated in one-back trials; and we excluded responses from one-back trials to have an equal number of responses for each face.

We recorded from implanted depth electrodes in the amygdala and hippocampus from patients with pharmacologically intractable epilepsy. Bipolar wide-band recordings (0.1–9000 Hz), using one of the eight microwires as reference, were sampled at 32 kHz and stored continuously for off-line analysis with a Neuralynx system. The raw signal was filtered with a zero-phase lag 300–3000 Hz band-pass filter and spikes were sorted using a semi-automatic template matching algorithm as described previously^72^. Units were carefully isolated, and recording and spike sorting quality were assessed quantitatively. Only units with an average firing rate of at least 0.15 Hz (entire task) were considered. Only single units were considered. Trials were aligned to stimulus onset and we used the mean firing rate in a time window 250 ms to 1000 ms after stimulus onset as the response to each face.

### Pairwise distances in the face space

We employed a pairwise distance metric^19^ to compare neural coding of face identities between primate neurons and DNN units. For each pair of identities, we used the dissimilarity value (1−Pearson’s *r*)^73^ as a distance metric. The primate neuronal distance metric was calculated between firing rates of all recorded neurons and the DNN distance metric was calculated between feature weights of all DNN units. We then correlated the primate neuronal distance metric and the DNN distance metric. To determine statistical significance, we used a nonparametric permutation test with 1000 runs. In each run, we randomly shuffled the face labels and calculated the correlation between the primate neuronal distance metric and the DNN distance metric. The distribution of correlation coefficients computed with shuffling (i.e., null distribution) was eventually compared to the one without shuffling (i.e., observed response). If the correlation coefficient of the observed response was greater than 95% of the correlation coefficients from the null distribution, it was considered significant. A significant correlation indicated that the DNN face space corresponded to the primate neuronal face space^19^. We computed the correlation for each DNN layer so that we could determine the specific layer that the neuronal population encoded. For each face identity, we averaged the response of all faces of that identity to get a single mean firing rate. To get temporal dynamics, for human neurons, we used a moving window with a bin size of 500 ms and a step size of 50 ms. The first bin started −300 ms relative to trial onset (bin center was thus 50 ms before trial onset), and we tested 19 consecutive bins (the last bin was thus from 600 ms to 1100 ms after trial onset). We used false discovery rate (FDR)^35^ to correct for multiple comparisons across DNN layers or time bins. For monkey neurons, we used a moving window with a bin size of 40 ms and a step size of 10 ms. The first bin started −70 ms relative to stimulus onset (bin center was thus 50 ms before stimulus onset), and we tested 26 consecutive bins (the last bin was thus from 180 ms to 220 ms after stimulus onset). We used Bonferroni correction to correct for multiple comparisons across DNN layers or time bins.

### Statistics and reproducibility

To select identity-selective units, we used a one-way ANOVA to identify identity-selective units that had a significantly unequal response to different identities (*P* < 0.01; Supplementary Fig. 2a). We further imposed an additional criterion to identify a subset of identity-selective units with selective identities (Supplementary Fig. 2a): the response of an identity was 2 standard deviations (SD) above the mean of responses from all identities. These identified identities whose response stood out from the global mean were the encoded identities. We refer to the units that encoded a single identity as single-identity (SI) units and we refer to the units that encoded multiple identities as multiple-identity (MI) units.

We followed the identical selection procedure for primate neurons. We used the mean firing rate in a time window 250–1000 ms after stimulus onset as the response to each face for primate neurons. Note that we also used this response to study the correlation between DNN units and primate neurons.

To select DNN feature units, we employed the same procedure as we did with human neurons^20^. We first estimated a continuous spike density map in the feature space by smoothing the discrete activation map using a 2D Gaussian kernel (kernel size = feature dimension range * 0.2, SD = 4). We then estimated statistical significance for each pixel by permutation testing: in each of the 1000 runs, we randomly shuffled the labels of faces. We calculated the *P* value for each pixel by comparing the observed spike density value to those from the null distribution derived from permutation. We lastly selected the region with significant pixels (permutation *P* < 0.01, cluster size >0.23 * pixel number in the whole space). We also applied a mask to exclude pixels from the edges and corners of the spike density map where there were no faces because these regions were susceptible to false positives given our procedure. If a unit had a region with significant pixels, the unit was defined as a feature unit and demonstrated region-based feature coding. We selected feature units for each individual DNN layer.

### Reporting summary

Further information on research design is available in the Nature Research Reporting Summary linked to this article.

## Supplementary information

Reporting Summary

Supplementary Information

Supplementary Data 1

Supplementary Data 2

Supplementary Data 3

Supplementary Data 4

Supplementary Data 5

Supplementary Data 6

Supplementary Data 7

Description of Additional Supplementary Files

## Data Availability

The full dataset for this study is publicly available on OSF (https://osf.io/824s7). The source data to generate each figure is available from Supplementary Data 1–7.

## Code availability

The source code for this study is publicly available on GitHub (10.5281/zenodo.6575224).
