# Supplementary material for: Face identity coding in the deep neural network and primate brain
Source: Commun Biol. 2022 Jun 20;5:611. doi: 10.1038/s42003-022-03557-9 (PMC9209415; doi:10.1038/s42003-022-03557-9)
Supplement: Supplementary file 2 — Supplementary Information [file 42003_2022_3557_MOESM2_ESM.pdf]

Supplementary Information

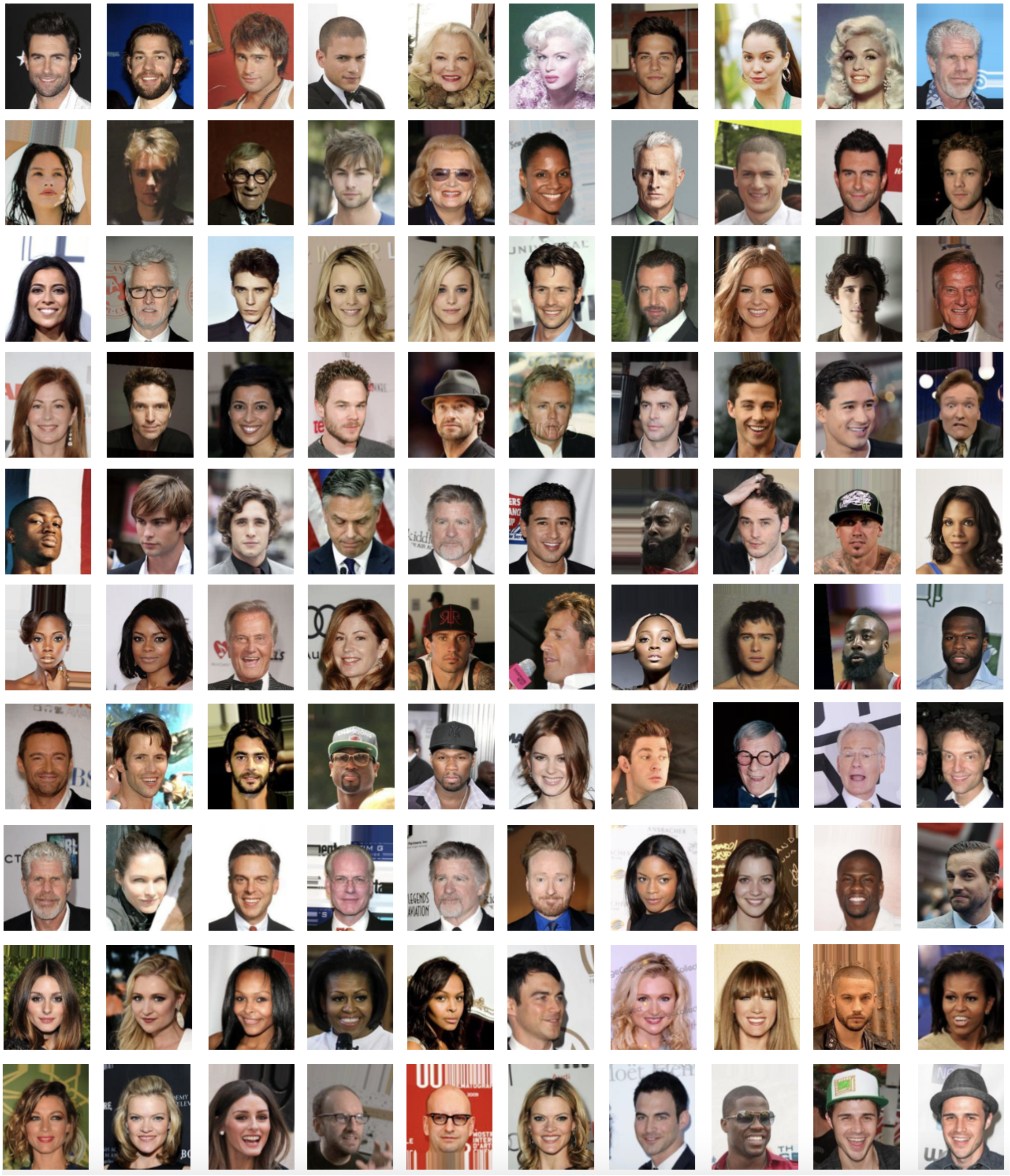

Supplementary Fig. 1. Sample stimuli. Faces from 50 celebrity identities were used for neural recordings. The identities were diverse in race, gender, and age, with a variety of facial

expressions. All images had the same resolution, and the faces had a similar size and position in the images.

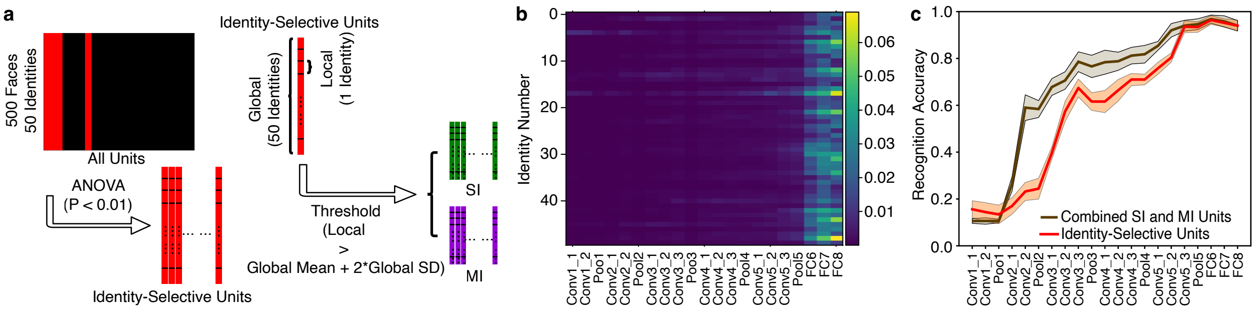

Supplementary Fig. 2. Additional results for identity-selective DNN units. **(a)** Procedure for selecting identity-selective DNN units. We used a one-way ANOVA to identify identity-selective DNN units that had a significantly unequal response to different identities ($P < 0.01$). We further imposed an additional criterion to identify a subset of identity-selective DNN units with selective identities: the response of an identity was 2 standard deviations (SD) above the mean of responses from all identities. These identified identities whose response stood out from the global mean were the encoded identities. We refer to the DNN units that encoded a single identity as single-identity (SI) units and we refer to the DNN units that encoded multiple identities as multiple-identity (MI) units. **(b)** The encoding frequency of each face identity in each deep neural network (DNN) layer. Color coding indicates the encoding frequency of each DNN layer (i.e., the number of DNN units encoding an identity divided by the total number of DNN units of a layer). **(c)** Combined SI and MI units demonstrated even better discriminability of face identities than identity-selective units. Identity recognition accuracy is shown for each DNN layer. Error shade denotes one standard deviation across 5-fold cross validation. Brown: combined SI and MI units. Red: identity-selective units.

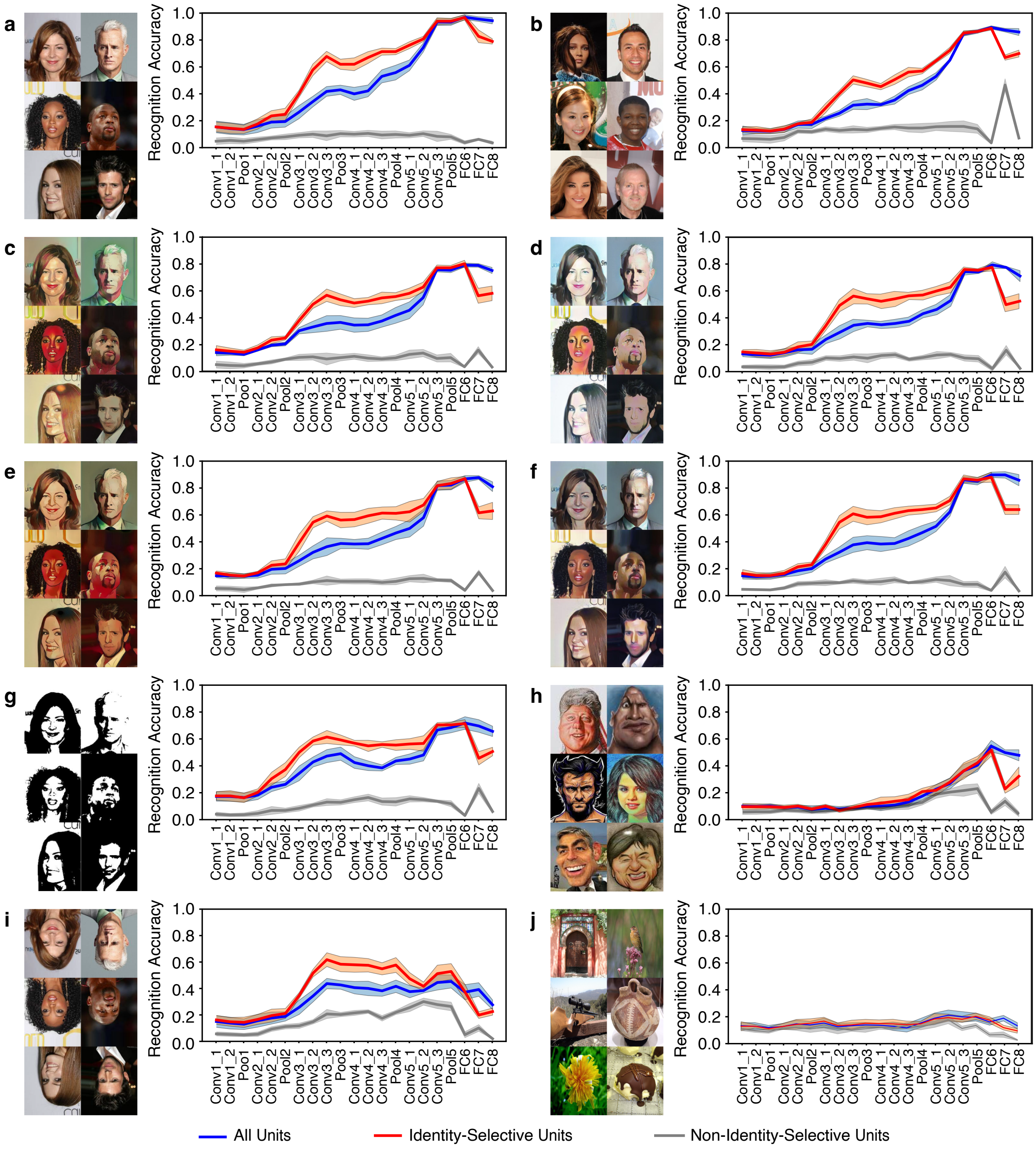

Supplementary Fig. 3. Recognition accuracy when we equated the number of identity-selective units and non-identity-selective units per layer. **(a)** Original faces used to identify identity-selective units. **(b)** Faces from a different set of 50 identities randomly selected from the CelebA database. **(c)** Original faces in the cartoon style Hayao. **(d)** Original faces in the cartoon style Hosoda. **(e)** Original faces in the cartoon style Paprika. **(f)** Original faces in the cartoon style

Shinkai. **(g)** Original faces in the Mooney style. **(h)** A different set of celebrity caricature faces. **(i)** Original faces in inversion. **(j)** A set of non-face objects selected from the ImageNet stimuli. Identity recognition accuracy is shown for each deep neural network (DNN) layer. Error shade denotes one standard deviation across 5-fold cross validation. Blue: all units from each DNN layer. Red: identity-selective units. Gray: non-identity-selective units.

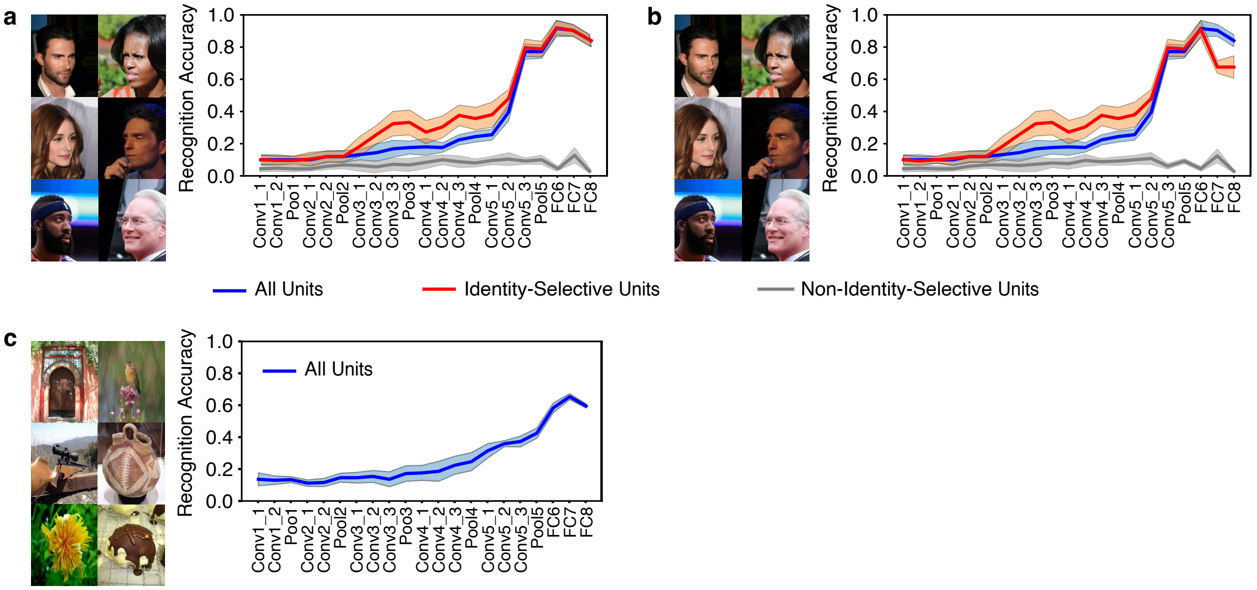

Supplementary Fig. 4. Recognition accuracy for profile faces and non-face objects. **(a, b)** Profile faces. For each face identity in **Fig. 2a**, we selected 5 profile faces from the CelebA dataset for analysis. **(a)** Analysis with all identity-selective and non-identity-selective units. **(b)** Analysis with equal number of identity-selective units and non-identity-selective units per layer. **(c)** Non-face objects. Legend conventions as in **Supplementary Fig. 3**.

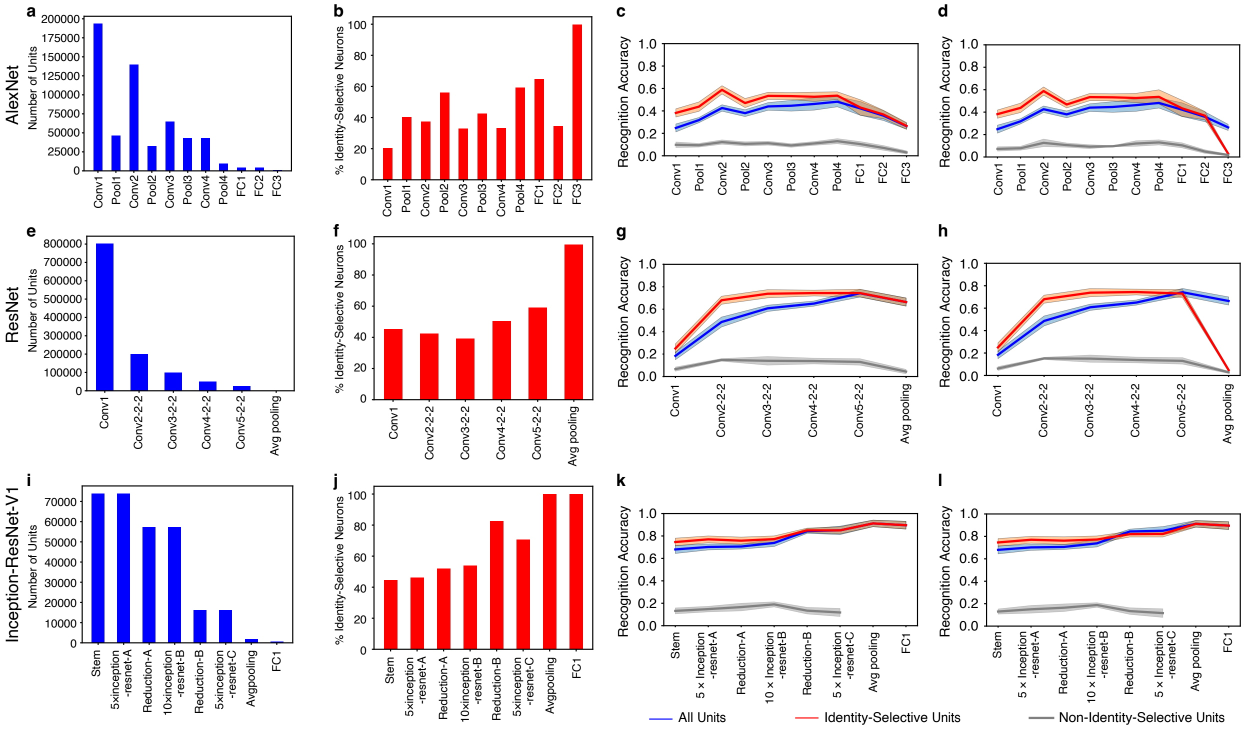

Supplementary Fig. 5. Recognition accuracy for different DNNs. **(a-d)** AlexNet [1] pre-trained by images from ImageNet [2]. **(e-h)** ResNet [3] pre-trained by images from MS-Celeb-1M [4]. **(i-l)** Inception-ResNet-V1 [5] pre-trained by images from CASIA-WebFace [6]. No fine-tuning was applied to these DNNs. **(a, e, i)** Number of neurons per layer. **(b, f, j)** Percentage of identity-selective neurons per layer. Note that all units in the last two layers of Inception-ResNet-V1 were identity-selective units. **(c, g, k)** Recognition accuracy. **(d, h, l)** Recognition accuracy with equal number of identity-selective units and non-identity-selective units per layer. Legend conventions as in **Supplementary Fig. 3**.

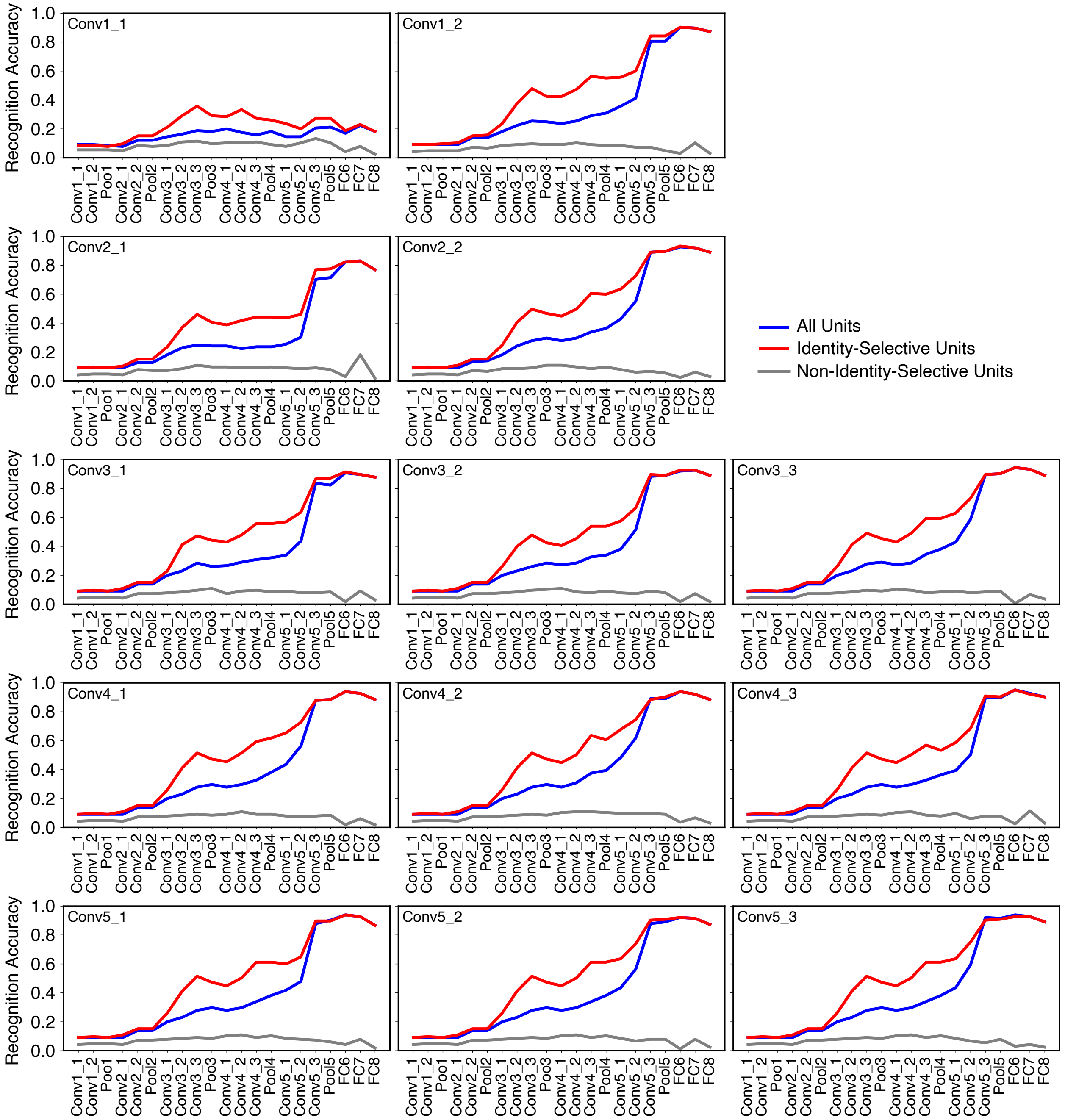

Supplementary Fig. 6. Recognition accuracy following a random dropout of a single layer of DNN units. 30% of the DNN units were dropped. The label in each plot indicates the layer that dropout was performed. Legend conventions as in **Supplementary Fig. 3**.

Supplementary References

1. Krizhevsky, A., I. Sutskever, and G.E. Hinton, ImageNet classification with deep convolutional neural networks, in Proceedings of the 25th International Conference on Neural Information Processing Systems - Volume 1. 2012, Curran Associates Inc.: Lake Tahoe, Nevada. p. 1097-1105.
2. Deng, J., et al. Imagenet: A large-scale hierarchical image database. in 2009 IEEE conference on computer vision and pattern recognition. 2009. Ieee.
3. He, K., et al. Deep residual learning for image recognition. in Proceedings of the IEEE conference on computer vision and pattern recognition. 2016.
4. Guo, Y., et al. Ms-celeb-1m: A dataset and benchmark for large-scale face recognition. in European conference on computer vision. 2016. Springer.
5. Szegedy, C., et al. Inception-v4, inception-resnet and the impact of residual connections on learning. in Thirty-first AAAI conference on artificial intelligence. 2017.
6. Yi, D., et al., Learning face representation from scratch. arXiv preprint arXiv:1411.7923, 2014.
