# Supplementary material for: Face identity coding in the deep neural network and primate brain
Source: Commun Biol. 2022 Jun 20;5:611. doi: 10.1038/s42003-022-03557-9 (PMC9209415; doi:10.1038/s42003-022-03557-9)
Supplement: Supplementary file 10 — Description of Additional Supplementary Files [file 42003_2022_3557_MOESM10_ESM.pdf]

Description of Additional Supplementary Files

File name: Supplementary Data 1
Description: Source data for generating Fig. 1.

File name: Supplementary Data 2
Description: Source data for generating Fig. 2.

File name: Supplementary Data 3
Description: Source data for generating Fig. 4.

File name: Supplementary Data 4
Description: Source data for generating Fig. 5.

File name: Supplementary Data 5
Description: Source data for generating Fig. 6.

File name: Supplementary Data 6
Description: Source data for generating Fig. 7.

File name: Supplementary Data 7
Description: Source data for generating Fig. 8.
